# Mediating role diet self-efficacy plays in the relationship between social support and diet self-management for patients with type 2 diabetes

**DOI:** 10.1186/s13690-021-00533-3

**Published:** 2021-01-31

**Authors:** Li Yang, Kun Li, Yan Liang, Qiuli Zhao, Dan Cui, Xuemei Zhu

**Affiliations:** 1 School of Nursing, Qingdao University, 15 Ningde Road, Qingdao, Shandong 266071 People’s Republic of China; 2 Department of Statistics, The 2nd Affiliated Hospital of Harbin Medical University, 246 XueFu Road, Harbin, 150086 Heilongjiang Province China; 3 School of Nursing, Zhejiang Chinese Medical University, Hangzhou, 310053 Zhejiang Province China; 4 School of Nursing, Harbin Medical University, Harbin, 150086 Heilongjiang Province China; 5 Department of Nursing, The 2nd Affiliated Hospital of Harbin Medical University, Harbin, 150086 Heilongjiang Province China

**Keywords:** Diabetes mellitus, type 2, Self efficacy, Diet, Self-management, Social support

## Abstract

**Background:**

It has previously been established that patients who have strong barriers to their diet self-management are more likely to have weak social support; however, the key mechanisms underlying the association between these two variables have not yet been established. This study aims to examine the potential role that diet self-efficacy plays in the relationship between social support and diet behavior in patients with type 2 diabetes mellitus (T2DM).

**Methods:**

It was a cross-sectional survey. Three hundred-eighty patients diagnosed with T2DM were recruited for this study from five community health centers in China. The Chronic Disease Resource Scale (CIRS), Cardiac Diet Self-efficacy Scale (CDSE), and Food Control Behavior Scale (FCBS) were used to estimate participants’ utilization of social resources, diet self-efficacy, and diet self-management, respectively. The data were analyzed utilizing structural equation modelling.

**Results:**

The results suggest that both higher levels of social support and diet self-efficacy are related to higher levels of diet self-management. The mediating effect that diet self-efficacy has on the relationship between social support and diet self-management was significant (β = .30, *p* < .05), explaining 55.68% of the total effect of social support on diet self-management.

**Conclusions:**

Diet self-efficacy plays a mediating role in the association between social support and diet behavior in patients with type 2 diabetes mellitus.

## Background

Diabetes, a chronic condition characterized by high blood glucose levels [1], is a global public health concern [2]. In 2017, global incidence, prevalence, death, and disability-adjusted life-years (DALYs) related to diabetes were 22.9 million, 476.0 million, 1.37 million, and 67.9 million, with a projection to 26.6 million, 570.9 million, 1.59 million, and 79.3 million in 2025, respectively [3]. In particular, type 2 diabetes mellitus (T2DM) is a major public health issue, and approximately 415 million adults have been diagnosed with T2DM worldwide. Moreover, the number is likely to increase to 642 million by 2040 if measures to address the condition are not taken [4]. In particular, in China, the prevalence of T2DM increased from 0.67 to 11.6% between 1980 and 2010, and there are no signs of this rise abating [5]; A large national survey showed that 12.8% of adults aged 18 and older living in mainland China had diabetes in 2017. And the total number of patients with diabetes in China is estimated to be 129.8 million [6]. The rise for the number is mainly due to the Chinese people’s diet structure (used to starchy foods), lifestyle, and aging population [7]. Diabetes is related to poor quality of life (QoL) and high morbidity and mortality, and diabetes has serious economic consequences for individuals, families, and healthcare systems. It is estimated that health expenditure in China relating to diabetes will reach 7.45–14 million US dollars by 2030 [8].

To combat the onset of T2DM, the predominant form of diabetes (> 95% of all cases), diet is considered to be an integral aspect [9]. However, diet self-management is not always effective. Specifically, one study revealed that only 23.5% of patients maintain long-term compliance with diet self-management [10]. Diet self-management behaviors for diabetes refer to a series of complex diet behaviors adopted by diabetic patients in order to successfully manage their chronic condition themselves [11]. Previous studies have shown that a patient’s adherence to diet treatment is affected by several factors, not only at the patient level (e.g., self-efficacy), but also at the provider or service level (e.g., organization of health services, social support) [12].

Social support refers to the perception and actuality of an individual receiving care and support and assistance when needed from family members, relatives, colleagues, organizations, and their community [13]. Many experimental studies have described the effect social support interventions have on the self-management of T2DM patients [14–16]. For example, individuals who receive such support can obtain advice on means of coping with related difficulties and can enjoy positive communication concerning diabetes care. However, several studies have shown that the variable of social support is challenging to study, and it has been speculated that, for T2DM, patients’ social support levels represent an indirect factor that affects compliance with diet control [17, 18]. Furthermore, a considerable body of evidence has revealed that there is a relationship between social support and diet self-management, yet the mechanisms underlying the association between social support and diet self-management remain unclear.

Self-efficacy is a critical concept in social cognitive theory [19], and it is defined as an individual’s confidence in his or her ability to conduct behaviors that will achieve desired outcomes. Meanwhile, diet self-efficacy refers to the degree to which individuals have “confidence that they can use the skills they possess to achieve healthy eating behaviors” [20]. Centis et al. [21] show that diet self-efficacy is significantly related to increased diet-promoting behaviors. In fact, various studies have shown that T2DM educational programs applying self-efficacy theory could improve self-management [22, 23], and can delay the occurrence of complications arising from the condition [24].

Although previous studies have revealed relationships between social support and diet self-management, the mechanisms underlying these associations are unknown. Considering this, the present study seeks to delineate the process by which social support relates to diet self-management by examining diet self-efficacy as a mediator of this association. Such identification of potential mediators could facilitate their use as important intervention targets for improving the diet self-management behaviors of T2DM patients. Specifically, we hypothesize that social support is related both to diet self-efficacy and diet self-management, and that diet self-efficacy has a mediating effect on the association between social support and diet self-management.

The Predisposing, Reinforcing, and Enabling Constructs in Educational Diagnosis and Evaluation (PRECEDE), which was established by Lawrence Green, is an effective theoretical model for community analysis and program development [25]. PRECEDE is a planning model for health promotion programs [26], and it provides a framework in which predisposing (self-efficacy), reinforcing (e.g., influences of others, family, peers, and health professionals), and enabling factors (e.g., availability of resources and skills) are considered as the factors that are most likely to impact behavior [27]. Thus, in the present study, we consider predisposing, reinforcing, and enabling factors that could affect diet self-management in patients with T2DM. In the current study, social support for diabetes is defined as relating to the reinforcing and enabling constructs (external factors) that influence patients’ diet behaviors and diet self-efficacy concerns predisposing constructs (internal factors). These definitions are in accordance with the PRECEDE model.

**Fig. 1:**
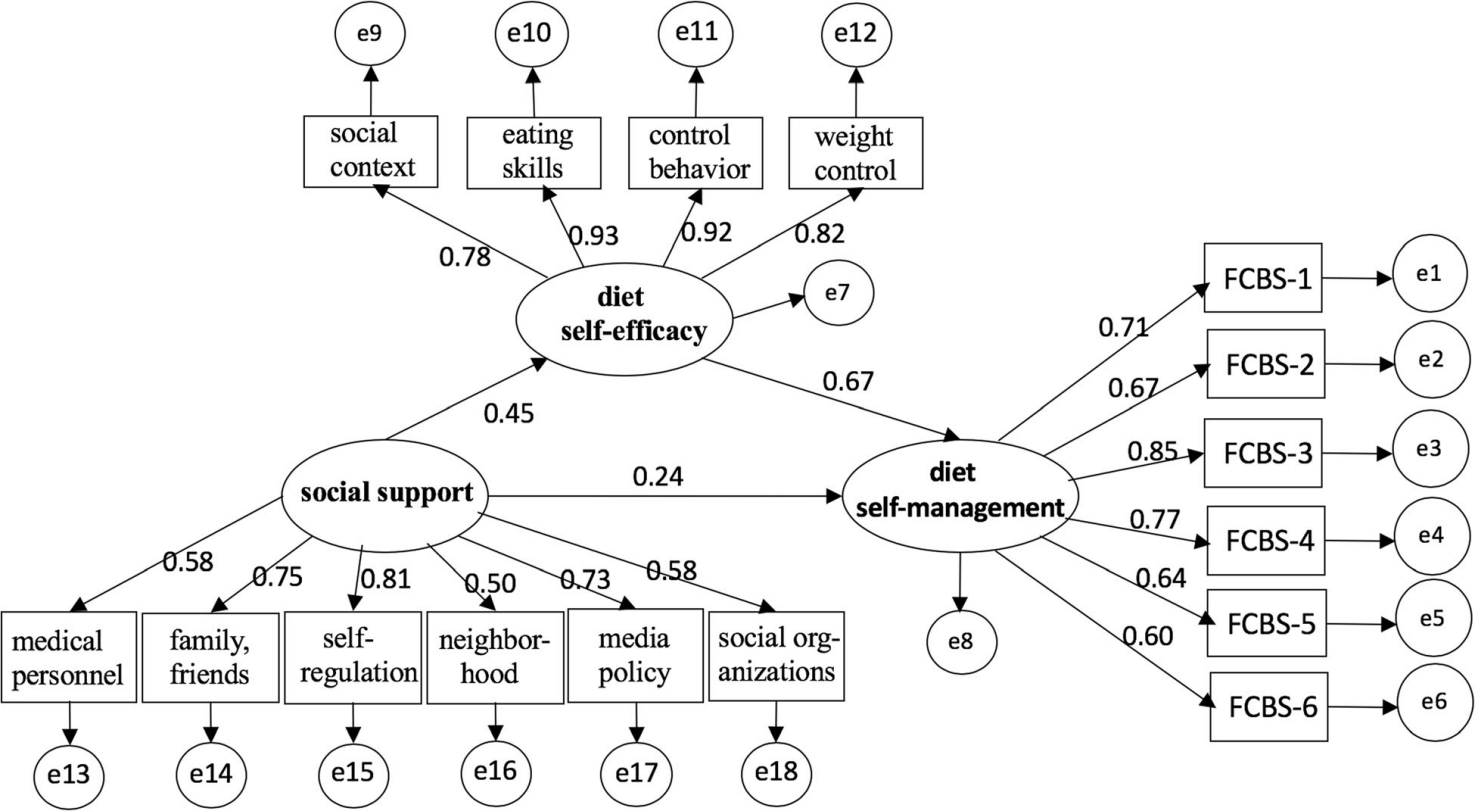
Structural equation model with indirect and direct effects of Diet Self-efficacy and Social Support on Diet Self-management in Patients with Type 2 Diabetes, China, 2017 (*n* = 358)

Planners may regard determinants as change processes that must be activated or initiated if the necessary behavioral and environmental changes have occurred [25]. Thus, in short, this study applies the PRECEDE model in an attempt to predict the determinants of the diet self-efficacy of T2DM patients by examining the relationship between social support in their communities and their diet behaviors. Based on the PRECEDE model and the above literature review, a hypothesized model has been created, and it is summarized in Fig. 1.

## Method

### Study design, sample, and procedure

To perform this examination, a cross-sectional survey was conducted from October 2016 to January 2017 in five community practice which located in Harbin, Heilongjiang Province of China. For the sample, inclusion criteria were: (1) having been diagnosed with T2DM at least one year prior to the study, (2) having the ability to perform self-management, and (3) taking medication for diabetes and in a stable condition. Meanwhile, exclusion criteria were: (1) possessing serious complications or comorbidities (diabetic nephropathy, diabetic retinopathy, diabetes with coronary heart disease, etc.), (2) possessing severe psychiatric disorders (ICD-10 codes: F01.1, F01.9, F03, F20.9, F25.9 and F29, 3) having received radiotherapy or chemotherapy within the last six months. All patients signed informed consent forms before participating in the study.

In order to ensure uniform data collection, data collectors were trained in survey content and investigation procedures. Next, we reviewed patients’ chronic illness records through their community profiles and evaluated their eligibility based on the inclusion and exclusion criteria. Next, we called eligible patients, explained the benefits and risks of the study, and we asked them to participate. We scheduled appointments for the patients who agreed to participate to complete the study questionnaire. At these meetings, the data collectors explained the aim of the study and verified each patient’s eligibility. The patients then signed informed consent forms, and they were permitted to retain a copy of their forms. Next, participants completed the study questionnaire. For patients who were unable to complete the questionnaire independently, the researchers read the survey questions aloud and marked the patients’ answers for each item. The entire investigation process took approximately 10–15 min. A total of 380 T2DM patients agreed to participate, and 358 completed responses were ultimately acquired (giving an effective response rate of 94.2%).

### Measurements

#### Socio-demographic characteristics

We collected the data about socio-demographic variables, including gender, age, marital status, education level, income (Chinese currency: RMB), duration of diabetes. In addition, complications or comorbidities were recorded, with 0(none),1–2 (1 or 2 kinds of complications or comorbidities) and ≥ 3(3 or more).

#### Social support

Participants’ social support was evaluated using the Chronic Disease Resource Scale (CIRS), which was developed in conformance with the Social Ecological Model created by Glasgow, Strycker, Toobert, and Eakin [28]. This 27-item tool collects data concerning seven support-related resources: medical personnel, family and friends, self-regulation, neighborhood communities, social organizations, media policy, and work environments. Responses to each item were scored using a five-point Likert scale that ranges from 1 (“not at all”) to 5 (“many” or “a considerable amount”). In cases where the average score for all items is less than 3, this indicates that, for that respondent, social resource utilization is not ideal. CIRS has been translated into many various language versions and has been used with a number of different populations (e.g., in Spain and Thailand), which shows that it has good psychological measurement characteristics [29]. The Cronbach’s α value of this survey was determined to be .93.

#### Diet self-efficacy

We also modified the Cardiac Diet Self-efficacy Scale (CDSE), which was developed by Hickey Owen, and Froman [30], and we used this to determine whether the patients possessed the necessary skills to have the level of the confidence in their healthy eating behaviors. It has previously been shown that this scale, which has relatively high reliability and validity, is appropriate for use with a number of chronic diseases [31, 32]. Specifically, the CDSE is a 16-item assessment instrument that measures four aspects of diet self-efficacy: diet in a social context, healthy eating skills, food control behavior, and weight control. Items are scored using a five-point Likert scale that ranges from 1 (“very low confidence”) to 5 (“very high confidence”), with higher scores revealing stronger diet self-efficacy. The scale has been determined to possess better internal consistency (Cronbach’s α = .86), test-retest reliability (.86), and behavioral predictive validity. For this survey, the Cronbach’s α is .95.

#### Diet self-management behavior

Next, we used the Food Control Behavior Scale (FCBS) to measure participants’ diet self-management behavior. This is a subscale (containing six items) of the Diabetes Self-management Behavior Scale developed by Wang et al. [33]. Here, items are scored using a five-point Likert scale that ranges from 1 (“never do”) to 5 (“always do”). The higher the score, the more frequently they engaged in diet self-management behaviors. The highest possible score is 30, with scores over 24 indicating good behavior and scores less than 12 indicating poor behavior; scores between 12 and 24 are considered average. The scale demonstrated accepted internal consistency (Cronbach’s α = .91) and better test-retest reliability (.83). The Cronbach’s α for this survey was determined to be .90.

### Data analysis

Statistical analyses were conducted utilizing SPSS version 21.0 (SPSS 21.0, Beijing, China), and structural equation modeling (SEM) was performed using AMOS 21.0. Before the analysis, we applied expectation maximization (EM) to impute missing data using SPSS’ missing value analysis. First, Pearson coefficients were used to evaluate the bivariate correlations between the variables, and then SEM was conducted to test the proposed model. SEM could examine the variance/covariance matrix obtained by the maximum likelihood estimation, and it was considered suitable to develop a model to explain the association between the study variables. The criteria utilized to examine the structural model were the chi-square (χ^2^)/df, Normed Fix Index (NFI), Incremental Fit Index (IFI), Confirmatory Fit Index (CFI), and the Root-Mean-Square Error of Approximation (RMSEA). Here, the indicator, which demonstrated an adequate fit of the data to the model, was a nonsignificant χ^2^,χ^2^/df ≤ 3, an RMSEA value from .05 to .08, and a NFI, IFI, and CFI value of >.90. Meanwhile, a good fit of the data was determined to be demonstrated by a RMSEA value of <.05 [34]. The NFI, IFI, and CFI values could range from 0 to 1, with 1 indicating a perfect data fit. Further, RMSEA values could also range from 0 to 1, with smaller values indicating a better fit. By examining the direction and magnitude of the path coefficient, the hypotheses of the specific structural associations of the variables in the model were evaluated. The path coefficient is a standardized regression coefficient (β) that represents the direct effect of a predictor variable on the response variable in the path model. In addition, variables with nonsignificant factor loadings were omitted from the SEM. Finally, a two-tailed α-value of .05 was set as the threshold for significance, and a power analysis was conducted. The result indicated that the sample size of 358 was adequate for this analysis to reach a power of > .80 [35].

## Results

### Participants

Demographic and disease characteristics are presented in Table 1. The mean age of the patients was 66.27 (SD: 10.33) years, and approximately half reported an education level of primary school or less (53.6%) and being of middle-income status (1000 to 3000 RMB; 64.80%). Furthermore, 46 participants (12.8%) lived alone and 173 (48.3%) received insulin treatment. The average duration with diabetes of the participants was 10.41 years (SD:6.80), while approximately 42.2% had experienced three or more complications or comorbidities.

**Table 1:** Frequency Distribution of Demographic Characteristics, Means, Standard Deviations of, Diet Self-management, in Patients with Type 2 Diabetes, China, 2017

Factors	n(%)	score	t/F	*P*
Sex			−3.90	*p* < .001
Male	165 (46.1)	19.05 ± 4.52		
Female	193 (53.9)	20.82 ± 4.05		
Age in years			23.27	*p* < .001
29–50	31 (8.7)	16.35 ± 4.90		
51–64	106 (29.6)	18.90 ± 4.17		
≥ 65	221 (61.7)	21.05 ± 4.00		
Marital Status			.46	*p* < .001
Married	312 (87.2)	20.41 ± 4.27		
Divorced/widowed/separated	46 (12.8)	19.95 ± 4.37		
Education level			1.59	.192
Junior high school or less	192 (53.6)	19.82 ± 4.06		
High school or Technical School	110 (30.7)	20.69 ± 4.39		
Junior College	35 (9.8)	19.11 ± 5.29		
Undergraduate or more	21 (5.9)	19.57 ± 4.93		
Income (average monthly, unit: RMB)			2.25	.082
<1000	47 (13.1)	18.70 ± 5.30		
1000–3000	232 (64.8)	20.35 ± 3.93		
3000–5000	65 (18.2)	19.95 ± 4.67		
>5000	14 (3.9)	18.86 ± 5.53		
duration			1.79	.168
≤ 5 years	100 (27.9)	19.36 ± 4.53		
5 years–10 years	114 (31.9)	20.03 ± 4.00		
>10 years	144 (40.2)	20.44 ± 4.47		
complications or comorbidities			2.49	.084
0	36 (10.0)	18.89 ± 4.50		
1–2	171 (47.8)	19.78 ± 4.54		
≥ 3	151 (42.2)	20.52 ± 4.05		

### Correlations between the study measures

Table 2 shows the correlations between the study variables. In light of the significant correlation between social support, potential mediators (diet self-efficacy) and diet self-management, a mediation test was performed subsequently.

**Table 2:** Mean, Standard Deviation, and Correlations between the Diet Self-efficacy, Social Support and Diet Self-management, in Patients with Type 2 Diabetes, China, 2017

Variables	1	2	3	M ± SD
1 Diet self-efficacy	1			54.60 ± 12.41
2 Social support	.42^*^	1		65.60 ± 19.25
3 Diet self-management	.74^*^	.23^*^	1	20.01 ± 4.36

*p < .05

Note. *M* mean, *SD* standard deviation

### The potential mediating role of diet self-efficacy

As shown in Table 3, social support has a direct effect on diet self-management (β = 0.24, *p* < .05), and has an indirect effect on diet self-efficacy. Further, diet self-efficacy has a significant mediating effect on the relationship between social support and diet self-management (β = 0.30), explaining 55.68% of the total effect of social support on diet self-management. Moreover, the hypothesized model indicated an adequate fit to the data (χ^2^/df = 2.17, GFI = 0.93, AGFI = 0.91, NFI = 0.90, IFI = 0.92, CFI = 0.92, RMSEA = .06). Figure 1 showed structural equation model with indirect and direct effects of diet self-efficacy and social support on diet self-management in patients with T2DM.

**Table 3:** Total, Direct, and Indirect Effects of the Social Support, Diet Self-efficacy on Diet Self-management, respectively, in Patients with Type 2 Diabetes, China, 2017

	Total effect	Direct effect	Indirect effects
Social support	.54	.24^*^	.30^*^
Diet self-efficacy	.67	.67^*^	–

*p < .05

## Discussion

The PRECEDE model recommends that the causes of health problems be analyzed from multiple perspectives, taking into account multiple determinants of diet behavior; however, very few previous studies have focused on the efficacy of the PRECEDE model in regard to T2DM. Thus, the present study is somewhat unique, as it applied the PRECEDE framework to test factors associated with the diet behaviors of individuals with T2DM.Our findings suggest that predisposing, enabling, and reinforcing factors are crucial for understanding and promoting diet behavior practices. In addition, we determined that diet self-efficacy mediates the association between social support and diet behavior; in other words, such a change in behavior requires both internal and external factors. The results also indicated that individuals with high levels of social support barriers tend to have low levels of diet self-efficacy, which in turn can lead to poor diet behavior.

Inducing factors are the factors that urge people to take necessary actions. Predisposing factors are factors that establish incentives to take a required behavior. In this study, predisposing factors specifically relate to diet self-efficacy. In the case of diabetes self-management, self-efficacy relates to a patient’s confidence in his or her ability to perform a variety of diabetes self-management behaviors.

Self-efficacy has two basic elements: efficacy expectations (self-efficacy) and outcome expectations [36]. Efficacy expectation refers to the confidence of individuals in their own behavioral ability and their confidence in their ability to overcome obstacles to achieve a certain goal. Outcome expectation refers to the belief of individuals that they will obtain a positive health outcome by performing a specific behavior [19]. Therefore, despite the obstacles in diet self-management, individuals with high levels of perceived diet self-efficacy will still attempt to achieve specific goals [37]. The current results reflect the positive impact of this model on predisposing factors (diet self-efficacy), which is consistent with previous studies that have found that it is a useful predictor of enhanced diabetes self-management [22].

Enabling factors contain the facilities and skills needed to change a behavior, while reinforcing factors increase the possibility of the continuation of the recommended behavior [38]. In this model, social support represents both the enabling factors and reinforcing factors. However, different from prior studies, it has produced a wide range of assessments of social support, discussing the utilization of medical personnel, family and friends, self-regulation, neighborhood communities, social organizations, media policy, and work environments. The results of our study demonstrate that social support plays a significant role in influencing, either directly or indirectly, changes in diet behavior.

Firstly, social support was found to be significantly and directly associated with increased diet-promoting behaviors. For instance, friends, family, and supporters of patients may provide information and tangible forms of support, and may set an example for healthy habits, thereby increasing the diet-promoting behaviors. Moreover, social support might have an emphasized role in terms of diet promotion, since individuals with diabetes can feel empowered when in a supportive social environment, which in turn could encourage them to engage in diet-promoting behaviors. Thus, for patients with diabetes, social support can constitute a fundamental approach to maintain self-management behaviors and overcoming barriers.

Furthermore, there are several possible explanations for the mediating role diet self-efficacy plays in the relationship between social support and diet self-management for diabetes patients. Self-efficacy is impacted by personal factors (i.e., age, education level) and environmental factors (i.e., barriers to behavior changes, social support). Further, King et al. [22] showed that self-efficacy was strongly associated with behavior-specific support from family, friends, and communities. Venkataraman et al. [39] found that positive family support increases peoples’ confidence in their ability to manage diabetes [15]. Another important finding is that, according to previous systematic reviews, the effectiveness of diabetes self-management programs is strongly associated with their duration, and the effectiveness may gradually disappear after the interventions end [40, 41]. Similarly, many studies have indicated that self-efficacy may be successful when initiating behavioral changes, but it has also been suggested that these changes may not be maintained in the long-term [42]. Thus, continued social support is key for long-term maintenance of self-efficacy [29]. If diabetes patients are to persist in diet self-management, social support may further strengthen the impact of dietary interventions, and social support may therefore help to promote enduring behavior change. Some previous studies that have examined dietary interventions [39, 40], but have not found significant improvements in their participants’ diet behaviors. However, many of these interventions concerned relatively intensive programs, and the course formats were rigid. Moreover, there has been some controversy suggesting that high-intensity education can lead to increased time and labor costs, increased feelings of burden, and subsequent negative effects on diet adherence [41]. Thus, a program with a rigid format may harm participants’ confidence and increase long-term dropout rates.

Public health professionals should not only pay attention to external environmental factors, such as the influence of social support on diet behavior of patients, but also attach great importance to the role of internal factors on diet behavior of patients with diabetes, especially patients’ diet self-efficacy. Therefore, in public health practice, professionals should design targeted intervention measures, so that individuals can improve their diet behaviors by enhancing their sense of self-efficacy.

This study has a number of strengths and weaknesses. The major strengths of this study are that it considers, based on the PRECEDE model, both internal and external factors that affect the diet self-management of T2DM patients. In addition, it is the first study to focus specifically on diet self-management. Furthermore, our research, for the first time, validates the role diet self-efficacy plays in the association between social support and diet self-management. Nevertheless, there are also several limitations to this study.

Firstly, this study was a cross-sectional investigation. Therefore, despite the hypothetical mediation model has a theoretical basis, it was not possible to draw firm conclusions about the directionality of the relationships between the study variables. Thus, longitudinal studies should be conducted to further understand the mediation model proposed in this study. Secondly, social support, diet self-efficacy and diet self-management behaviors were evaluated by self-report. So, these responses may be biased. However, this method is the only known feasible and cost-effective way to collect such data. Finally, we were unable to assign the participants randomly to the study; the respondents were selected by telephone follow-up. This means that the findings are probably not generalizable to all individuals with diabetes and should be interpreted as such.

## Conclusions

The results of this study highlight the mediating role diet self-efficacy plays in the association between social support and diet self-management. In essence, this study provides a starting point for future intervention studies on the effectiveness of implementation. Reflecting on patients’ diet self-efficacy can help to establish evidence-based and patient-centered interventional measures. Interventions that improve individuals’ self-efficacy of diabetes have the potential to remove barriers to social support and its benefits in regard to diet behavior. Moreover, our findings suggest that predisposing, enabling, and reinforcing factors are crucial for understanding diet-promoting behavior practices, which in turn may make contribution to better self-management and quality of life.

## Data Availability

The datasets used and/or analysed during the current study are available as well as by reasonable request to the corresponding author.

## Abbreviation

T2DM: Type 2 diabetes mellitus
PRECEDE: The Predisposing, Reinforcing, and Enabling Constructs in Educational Diagnosis and Evaluation
CIRS: Chronic Disease Resource Scale
CDSE: Cardiac Diet Self-efficacy Scale
FCBS: Food Control Behavior Scale
SEM: Structural equation modeling
EM: Expectation maximization
NFI: Normed Fix Index
IFI: Incremental Fit Index
CFI: Confirmatory Fit Index
RMSEA: Root-Mean-Square Error of Approximation
